# Speak Fast, Use Jargon, and Don’t Repeat Yourself: A Randomized Trial Assessing the Effectiveness of Online Videos to Supplement Emergency Department Discharge Instructions

**DOI:** 10.1371/journal.pone.0077057

**Published:** 2013-11-11

**Authors:** Clare L. Atzema, Peter C. Austin, Libo Wu, Michael Brzozowski, Michael J. Feldman, Michael McDonnell, Laurie Mazurik

**Affiliations:** 1 The Division of Emergency Medicine, Department of Medicine, University of Toronto, Toronto, Ontario, Canada; 2 Sunnybrook Health Sciences Centre, Toronto, Ontario, Canada; 3 The Institute for Clinical Evaluative Sciences, Toronto, Ontario, Canada; University of Pittsburgh Medical Center, United States of America

## Abstract

**Background:**

Emergency department discharge instructions are variably understood by patients, and in the setting of emergency department crowding, innovations are needed to counteract shortened interaction times with the physician. We evaluated the effect of viewing an online video of diagnosis-specific discharge instructions on patient comprehension and recall of instructions.

**Methods:**

In this prospective, single-center, randomized controlled trial conducted between November 2011 and January 2012, we randomized emergency department patients who were discharged with one of 38 diagnoses to either view (after they left the emergency department) a vetted online video of diagnosis-specific discharge instructions, or to usual care. Patients were subsequently contacted by telephone and asked three standardized questions about their discharge instructions; one point was awarded for each correct answer. Using an intention-to-treat analysis, differences between groups were assessed using univariate testing, and with logistic regression that accounted for clustering on managing physician. A secondary outcome measure was patient satisfaction with the videos, on a 10-point scale.

**Results:**

Among 133 patients enrolled, mean age was 46.1 (s.d.D. 21.5) and 55% were female. Patients in the video group had 19% higher mean scores (2.5, s.d. 0.7) than patients in the control group (2.1, s.d. 0.8) (p=0.002). After adjustment for patient age, sex, first language, triage acuity score, and clustering, the odds of achieving a fully correct score (3 out of 3) were 3.5 (95% CI, 1.7 to 7.2) times higher in the video group, compared to the control group. Among those who viewed the videos, median rating of the videos was 10 (IQR 8 to 10).

**Conclusions:**

In this single-center trial, patients who viewed an online video of their discharge instructions scored higher on their understanding of key concepts around their diagnosis and subsequent care. Those who viewed the videos found them to be a helpful addition to standard care.

**Trial Registration:**

ClinicalTrials.gov NCT01361932

http://clinicaltrials.gov/ct2/show/NCT01361932?term=nct01361932&rank=1

## Introduction

About a fifth of the population visits an emergency department each year [1]. The large majority of patients who visit the emergency department are subsequently discharged to their place of residence: only 13% are admitted to hospital [2]. For the 87% of patients who are discharged home, management does not end at emergency department departure; discharge instructions ensure appropriate ongoing self-care for the condition, direct follow-up care requirements, and delineate circumstances which mandate a return visit to the emergency department. The Centers for Medicare and Medicaid Services (CMS) support compliance measures for written discharge instructions in hospitalized patients [3]; emergency department discharge is not specifically addressed. 

Good discharge instructions may prevent subsequent hospitalizations [4,5] and lessen repeat emergency department visits in an already overwhelmed system; however, the time it takes to provide detailed discharge instructions to each and every patient seen in the emergency department may be prohibitive [6]. Emergency department crowding is ubiquitous around the world [7–10], and may further shorten the time spent with the doctor [7]: discharge instructions may be truncated in an effort to see more patients. In a hurried and unfamiliar environment, the patient’s ability to retain the instructions told to them by the doctor may be compromised. One study found that half of patients were deficient in their comprehension of what was told to them by the emergency physician, in particular in post-emergency department care [11]. Providing patients with common diagnoses with a website of vetted emergency department discharge instructions, where the patient can view a short video related to their discharge diagnosis, might remind patients of forgotten information as well as bridge gaps in instructions that were provided by the emergency physician. Patients could replay the instructions repeatedly if needed, in order to learn at their own pace [12]. 

The large majority of persons in the Western world now have access to online information [13]. We hypothesized that a multi-modal approach to the provision of emergency department discharge instructions, including the use of vetted online videos, would improve patient understanding of key aspects of care after discharge. We aimed to determine the effect of online videos on patient understanding and recall of their discharge instructions.

## Materials and Methods

### Study Design

This single-center, randomized controlled trial complied with the Declaration of Helsinki and was approved by the research ethics board at Sunnybrook Health Sciences Centre. All participants gave written informed consent. The protocol for this trial and supporting CONSORT checklist are available as supporting information; see Checklist S1 and Protocol S1. The trial was registered as NCT01361932 at clinicaltrials.gov.

### Study Setting

The study was set in the emergency department of Sunnybrook Health Sciences Centre, a tertiary adult hospital in Toronto with an annual census of 45,000. The hospital is a level 1 trauma center, with consultation services available for all major sub-specialties, including neurosurgery and vascular surgery. The site serves as the home of the University of Toronto emergency medicine residency program.

### Study Patients

Patients of any age who were discharged from the emergency department of the Sunnybrook Health Sciences Centre were eligible for the study if they had one of 38 final emergency department diagnoses. Discharge diagnoses were determined by the managing emergency physician, and were not dictated by the study. Patients could be referred to the research assistant by the managing emergency physician, or the research assistant could find them by reading patient triage notes and discharge diagnoses in the Emergency Department Information System (EDIS), the software that contains all registered emergency department patients (current and previous). Patients who did not speak English were eligible if their caregiver was able to speak fluent English, and agreed to watch the video for the patient, as well as answer the related questions. Similarly, parents of young children could participate on behalf of their child. Patients without access to the internet or to a telephone were excluded.

### Randomization Process

Based on the average number of eligible patients seen at the study site per day, and the a priori sample size calculation (see below), the research assistant was given a randomized schedule of 30 eight-hour shifts between November 7, 2011 and January 7, 2012, which included weekends. Shifts between midnight and 08:00 were not included. However if the patient presented overnight and was still in the emergency department when the research assistant arrived, the patient was approached by the research assistant for consent at that time. Consenting patients were randomized to the intervention or control group using simple randomization: a computer program was used to generate the random number sequence, and group assignment was kept in opaque numbered envelopes in the study emergency department. Patients who were randomized to watch a video were given a handout with the online site address and the name of the video with their diagnosis, and instructed to watch it within the next two days, in addition to usual care. Both groups were informed that they would be called and asked three questions pertaining to their discharge instructions. Patients were called by the research assistant between three and seven days after emergency department discharge; up to three attempts were made to contact the patient by phone, and email was also pursued if provided by the patient. In order to ask whether patients in the intervention group had viewed the video (in case they needed more time to watch it), the research assistant was not blinded to the patient’s group assignment. 

### Online Videos

Scripts for each of the 38 discharge diagnoses (Table 1) were created by the principal investigator. These were subsequently edited by four co-authors (MB, MJF, MM, LM) using a modified Delphi approach [14,15]. After two rounds, these co-authors met in person with the principal investigator to discuss outstanding suggestions and to finalize the scripts. All authors except PCA and LW are practicing emergency physicians from the study site, selected for the range of years in practice they represent (one had been in practice less than five years, two for five to 10 years, one for 10 to 20 years, and one more than 20 years). Scripts were pilot-tested on 10 laypersons for comprehension and adjusted as needed. Videos were recorded by the principal investigator and links were placed online on the hospital website (Sunnybrook.ca/eddischarge); videos ranged from three to six minutes in length.

**Table 1:** Available online videos of discharge instructions, by final emergency department discharge diagnoses.

	Final emergency department Diagnosis
1.	Abscess, Incision & Drainage
2.	Allergic reaction
3.	Ankle sprain
4.	Asthma exacerbation
5.	Atrial fibrillation
6.	Back strain
7.	Bell’s Palsy
8.	Broken bone, with splint
9.	Burns
10.	Cellulitis
11.	Croup
12.	Diverticulitis, uncomplicated
13.	Ear infection, inner - Otitis media
14.	Ear infection, outer - Otitis externa
15.	Eye scratch (corneal abrasion)
16.	Fever in a child
17.	Fingertip amputation
18.	Gastroenteritis, viral / vomiting and diarrhea
19.	Gout attack
20.	Head injury, minor, with concussion
21.	Head injury, minor, with return to play guidelines
22.	High blood pressure, out of control
23.	Kidney stone
24.	Laceration/cut, glue or tape used
25.	Laceration/cut, stitches or staples used
26.	Miscarriage, possible
27.	Nosebleed
28.	Palpitations
29.	Panic attacks
30.	Rib fracture or contusion (broken or bruised ribs)
31.	Sciatica
32.	Shingles
33.	Sore throat (pharyngitis)
34.	Tubal pregnancy, possible (ectopic pregnancy)
35.	Urinary retention
36.	Urinary tract infection
37.	Vertigo (peripheral) or “the spins”
38.	Whiplash/neck strain

### Discharge Questions

Based on the topics covered in the online videos, three key questions were created for each discharge diagnosis (Appendix S1). Questions were edited by the emergency physician co-authors using the modified Delphi technique, followed by a face-to-face meeting. Questions were pilot-tested on 10 laypersons for comprehension and adjusted as needed. Each question could be given half a point if a partial answer was given (specific answers worth partial scores were described a priori and are noted in Appendix S1), otherwise each correct answer was assigned one point. Inter-rater reliability of the test scores was performed on a subset of 30 patients by two co-authors (CLA and LW), and unweighted kappa values were utilized to determine agreement. 

### Outcome Measures

The primary outcome measure was the patient’s score (out of 3) on questions about their discharge instructions. The secondary outcome measure was patient rating (on a 10-point scale) of the value of the videos overall, and in specifically improving discharge instruction comprehension (both measured in patients who viewed a video).

### Data Analysis

The a priori-specified per protocol analysis of efficacy was a two-sample t-test comparing the mean score between the two treatment groups, using an intention-to-treat analysis. We also conducted a secondary analysis after discovering that the distribution of the scores in the intervention group was non-normal (left-skewed): we used the Kruskal-Wallis test to compare the distribution of scores between the intervention and control groups. In a sensitivity analysis, to ensure that clustering by physician did not change the results, we used regression modeling with generalized estimating equations (GEEs) to account for clustering by emergency physician. We dichotomized the outcome to create a binary outcome: a score of three versus a score of less than three. We then used logistic regression to estimate the effect of the intervention on the odds of receiving a score of three. The logistic regression model also adjusted for the four measured patient-level covariates: age, sex, whether English was the first language, and emergency department triage score (1 or 2 [highest acuity], 3, 4 or 5 [highest acuity], using the Canadian Triage and Acuity Scale [16]). The a priori-specified per protocol analysis for the secondary outcome measure was median with interquartile range (IQR). All analyses were done with SAS software (Version 9.2, SAS Institute Inc., Cary, NC).

### Power Calculation

Based on the per-protocol analysis, 63 patients per study arm were required for a two-sample t-test to have 80% power to detect a mean difference in tests scores of at least 0.5 points between study arms. This was based on the assumption that the standard deviation of the test score was 1.0 within each study arm and a type I error rate of 0.05.

## Results

**Figure 1:**
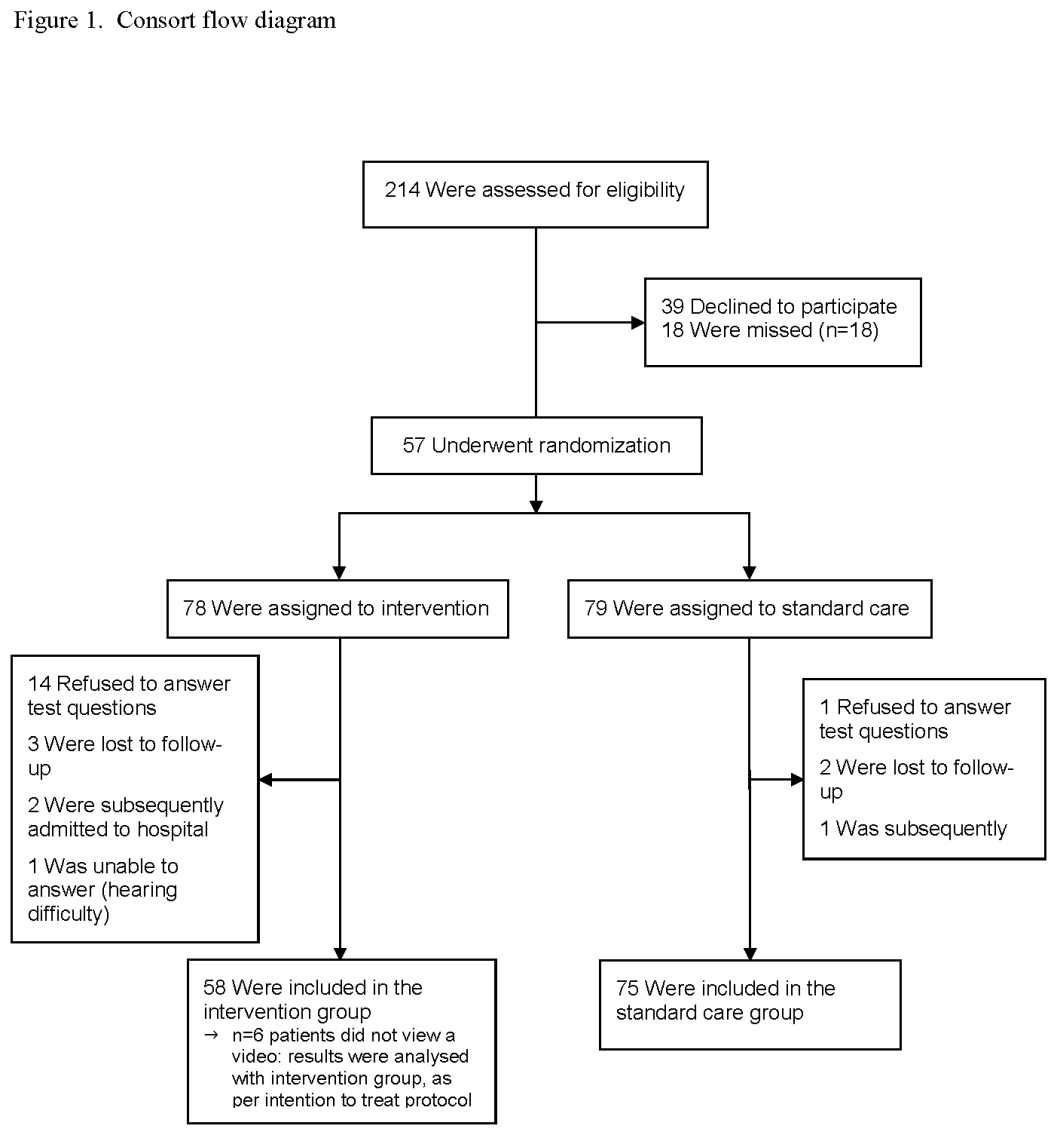
CONSOSRT flow diagram.

Enrollment and patient flow is shown in Figure 1. Excluded patients were not statistically different from those who were enrolled by age (p=0.12), sex (p=0.47), or triage score (p=0.44). Among the 133 patients enrolled, more patients in the intervention group refused to answer the study questions (and therefore were “lost to follow-up”, since no measurable answers were provided) than in the control group, resulting in 58 patients in the intervention group, and 75 in the control group. Patients lost to follow-up were not statistically different from those who completed follow-up by age (p=0.81), sex (p=0.49), or triage score (p=0.50). Among the 58 patients in the intervention group, six did not view the video, but did provide answers to the study questions; results from these patients were included in the intervention group, as per intention to treat principle.

Patient characteristics are shown in Table 2. Mean age of patients was 46.1 (s.d. 21.5), and 73 (54.9%) were female. The median triage score was 3, and English was the first language for 83.5% (95% confidence interval, 76.0 to 89.3) of patients. The most frequent final emergency department diagnoses were broken bone requiring a splint (19%) and cut requiring stitches (11%), consistent with other publications on procedures performed in the emergency department [17]. 

**Table 2:** Characteristics of 133 study patients.

Characteristic	All (n=133)	Intervention group (n=58)	Control group (n=75)	p
Age (s.d.)	46.1 (21.5)	48.5 (22.4)	44.3 (20.7)	0.26
Female sex	73 (54.9%)	35 (60.3%)	38 (50.7%)	0.27
ED Triage score, median*	3.0	3.0	3.0	0.30
English first language	111 (83.5%)	45 (77.6%)	66 (88%)	0.11
Final ED diagnosis				
Abscess, Incision & Drainage	6	3	3	
Allergic reaction	4	2	2	
Ankle sprain	7	1	6	
Atrial fibrillation	6	6	0	
Back strain	6	3	3	
Bell’s Palsy	1	1	0	
Broken bone, with splint	25	7	18	
Burns	3	1	2	
Cellulitis	8	4	4	
Cut, stitches or staples used	14	7	7	
Cut, glue or tape used	6	4	2	
Diverticulitis, uncomplicated	2	1	1	
Ear infection, inner – Otitis media	1	1	0	
Gastroenteritis, viral / vomiting & diarrhea	2	0	2	
Gout attack	1	0	1	
High blood pressure, out of control	4	1	3	
Kidney stone	4	1	3	
Miscarriage, possible	3	2	1	
Head injury, minor, with concussion	6	3	3	
Nosebleed	8	3	5	
Palpitations	1	1	0	
Rib fracture or contusion (broken or bruised ribs)	1	0	1	
Shingles	1	0	1	
Sciatica	2	1	1	
Sore throat (pharyngitis)	3	2	1	
Urinary retention	1	0	1	
Urinary tract infection	2	1	1	
Whiplash/neck strain	1	0	1	
Vertigo (peripheral) or “the spins”	3	1	2	

ED: emergency department

Using Canadian Triage and Acuity Scale, score 1 (highest acuity) to 5 (lowest acuity) [16]

The inter-rater reliability of the test questions was excellent (κ=0.87) [18]. Univariate test results are shown in Table 3. Patients in the intervention group had significantly higher mean scores (2.5, s.d. 0.8) than those in the control group (2.1, s.d. 0.7) (p=0.002). Median scores were also higher in the intervention group (3.0, IQR 2.0 to 3.0) than in the control group (2.5, IQR 1.5 to 3.0) (p=0.001). In the adjusted analysis, the odds of receiving a score of 3 were 3.5 (95% confidence interval, 1.7 to 7.2) times higher in the intervention group, compared to the control group (p<0.001) (Table 3). Thus the results of the sensitivity analyses were consistent with that of the a priori specified per-protocol analysis.

**Table 3:** Univariate and adjusted results of testing of understanding of discharge instructions.

**Univariate Analyses**	**Score / 3**		**p**
Intervention group, mean (s.d.)	2.5 (0.7)		0.002
Control group, mean (s.d.)	2.1 (0.8)		
Intervention group, median (q1 – q3)	3.0 (2.0-3.0)		0.001
Control group, median (q1 – q3)	2.0 (1.5-3.0)		
Logistic Regression Model*, regressed on all answers correct (score of 3)	**adjusted ORs**	**95% CI**	**p**
Intervention group	3.5	1.72 - 7.23	<0.001
Age (per decade increase)	0.93	0.85 - 1.01	0.39
Female sex	1.19	0.63 - 2.25	0.59
High acuity triage (1/2)	1.38	0.65 - 2.92	0.40
Low acuity triage (4/5)	1.30	0.64 - 2.66	0.47
English as first language	1.13	0.35 - 3.60	0.84

SD: standard deviation; q: quartile; OR: odds ratio

* Hosmer and Lemeshow Goodness of fit test: Chi-square=10.74 / DF=8 / p=0.22

For the secondary outcome measure, patients who viewed a video gave it an overall median rating of 10 (IQR 8 to 10) and mean of 9.1 (s.d. 1.1). For improving their understanding of their discharge instructions, the median rating was also 10 (IQR 8 to 10), mean 9.0 (s.d. 1.5).

## Discussion

Comprehension is the major predictor of compliance with discharge instructions [19], and compliance with discharge instructions has been associated with better patient outcomes [4,5]. In this study we found that patients who watched an online video of targeted discharge instructions were more likely to comprehend and remember all of the key concepts in their discharge instructions, compared to those who received standard care. While the content of the videos can be altered with changes in practice guidelines, and to suit local patient characteristics and practice patterns, we found that the likelihood of a patient recalling all that we, as practicing emergency physicians, wanted them to know was significantly higher if they viewed an online video. Many studies have shown that the majority of patients leaving the emergency department are deficient in their understanding of one or more aspects of their discharge instructions [11,20]; our study demonstrates that patients can learn and recall all of the key concepts of their instructions, if those concepts are placed in an accessible video format.

As the population ages and emergency department crowding worsens [21,22], innovations are needed to minimize the effect of crowding on time spent explaining discharge instructions to patients [7]. Crowding is known to affect academic centers in particular [10], where discharge instructions have been shown to average 76 seconds [6]. In that study, information on diagnosis, expected course of illness, self-care, use of medications, time-specified follow-up, and symptoms that should prompt return to the emergency department were each discussed less than 65% of the time. Our study suggests that utilizing technology to deliver information on common discharge diagnoses is one way to offset communication deficiencies related to lack of time, in order to achieve patient understanding and retention of key discharge instruction concepts. 

Previous studies have shown that other modes of instruction, in addition to verbal instructions, can improve patient comprehension of discharge instructions, including illustrations [23] and written instructions [24,25]. Some emergency departments subscribe to a service that provides written, standardized discharge instructions, such as Exit-writer™. These may be very helpful if (a) the hospital subscribes to them, and (b) the managing emergency physician or nurse takes the time to print them out for each patient. However the locus of control for the provision of written emergency department discharge instructions lies with the busy emergency department staff, not the patient. In addition, depending on the patient population and their associated reading level, some patients may not fully comprehend written instructions [20,23].

Some studies have utilized videos to advise patients in the emergency department waiting room about expected emergency department course [26], or shown mobile videos to patients before leaving the emergency department [27], but to our knowledge none have provided open assess to diagnosis-specific videos that can be viewed from the patient’s home. The improvement in comprehension found in our study is logical given that repetition is key to learning [12], and that patients may re-play the video as often as needed in their home-setting. In addition, use of medical terminology, which has been shown to be the greatest contributor to poor comprehension of discharge instructions [28], is avoided in online videos, which may have further improved patient comprehension.

Patient satisfaction is strongly correlated with quality of discharge instructions received [29]. Therefore if hospitals aim to improve patient satisfaction, yet still see high volumes of patients, use of online discharge instruction videos may aid in reconciling these two goals. However hospitals may be dissuaded by the time and cost required to make vetted videos, which may be the greatest limitation to providing online discharge instructions. In addition, videos must be updated as new evidence alters post-emergency department management. The videos created for this study are freely available from our hospital website and can be utilized for the 38 discharge diagnoses listed.

It is not surprising that not all patients appeared to find the online videos useful, which was apparent from the loss to follow-up in the video group. Some of these patients indicated that they had not viewed the video, and therefore did not want to answer the questions (often they indicated that they would, but they did not get around to it and then stopped answering questions and/or phone calls). While some patients will find the videos helpful, others will have been seen in the emergency department on days when crowding was not an issue, thereby allowing the emergency physician more time to explain instructions in detail, and to answer patient questions. In addition, some patients will comprehend their discharge instructions fully the first time they are presented with them, particularly those who learn best via verbal instruction (as opposed to visual or written instruction). The videos are clearly a supplement to standard delivery of discharge instructions, and it is expected that not all patients will benefit from them. 

### Limitations

This study was conducted at a single site, which serves a slightly lower proportion of patients with a low socioeconomic status. Thus while we found that almost all patients had access to the internet, including older patients, this may not be the case in other areas. We did not assess educational level, although patients were randomized so this should limit possible bias. The research assistant was not blinded to group; while that was the original intention, we found that it was not feasible as some patients had not viewed the video by day three after discharge, and requested more time to view it. Results of our study may be partly driven by the discharge instructions for broken bone with splint and laceration/cut with stitches or staples, although together these diagnoses accounted for less than a third (30%) of the study patients.

## Conclusions

In the emergency department setting, online videos of diagnosis-specific discharge instructions improve patient comprehension and retention of key discharge details. Particularly in light of emergency department crowding and shortened doctor-patient interactions times, this tool should be offered to patients to supplement standard care.

## Supporting Information

Appendix S1 **Standardized telephone interview questions.**
(DOCX)

Checklist S1 **CONSORT checklist.**
(DOC)

Protocol S1 **Trial protocol.**
(DOC)
