# Supplementary material for: Speak Fast, Use Jargon, and Don’t Repeat Yourself: A Randomized Trial Assessing the Effectiveness of Online Videos to Supplement Emergency Department Discharge Instructions
Source: PLoS One. 2013 Nov 11;8(11):e77057. doi: 10.1371/journal.pone.0077057 (PMC3823877; doi:10.1371/journal.pone.0077057)
Supplement: Appendix S1 — Standardized telephone interview questions. (DOCX) [file pone.0077057.s001.docx]

Appendix S1. Standardized telephone interview questions

As you know, you were diagnosed with a ___ when you were in the ER. I am going to ask you three questions about your discharge instructions.

1. Abscess incision & drainage
   1. If there is drainage (fluid) coming from the wound, getting your bandages dirty, is this good or bad?
      1. Good (it is draining)
   2. Why is the cotton gauze put inside the wound?
      1. To keep the wound open, so it can drain.
   3. How long should you keep the packing in?
      1. 2 days (1 day is fine too, as is 3 days).
2. Allergic Reaction
   1. When should you use your Epi-pen?
      1. Tongue, mouth, or throat swelling, or difficulties breathing
   2. How long do you need to take diphenhydramine, or Benedryl, for, after you leave the ER? This includes non-drowsy versions of this medication, like “Reactine” or “Claritin”.
      1. 48 hours, or 2 days
   3. If you are uncertain whether you need it, should you use your Epi-pen?
      1. Yes
3. Ankle Sprain
   1. How do you decide when you can walk on your ankle?
      1. If it hurts a lot, don’t. (listen to your ankle, let your ankle tell you, etc)
   2. What is the most important position for your ankle or leg when you are recovering?
      1. Elevated / Up
   3. How long will it take to heal?
      1. Weeks (2 weeks or less, zero points). Up to 6 months to heal completely (only need to say ‘weeks’ or 3 weeks and up to get a point)
4. Asthma Exacerbation
   1. Which puffer should you take when your breathing and/or wheezing gets worse, during an asthma attack?
      1. Blue puffer / Ventolin puffer
   2. How often and how much should you be taking the blue puffer (Ventolin) in the first few days after you are discharged from the ER?
      1. 2 puffs (‘a few’ puffs is okay too) every 4 hours (or every 6 hours) – ½ pt each. “A lot” = ½ point
   3. When should you come back to the ER?
      1. Requiring > 2 puffs less than every 4 hours, can’t speak full sentences, or breathing labored.
5. Atrial Fibrillation
   1. What is the big concern about atrial fibrillation? What can happen to patients who have atrial fibrillation?
      1. A stroke
   2. If you were put on warfarin, does it matter which pharmacy you take the prescription to?
      1. Yes. Lots of interactions with warfarin
   3. OR
   4. [if not put on warfarin, as the following question] How is a stroke prevented?
      1. Aspirin or warfarin
   5. If you were put on a new medicine, such as aspirin, warfarin, or a rate control medicine, how soon do you need to see your family doctor?
      1. Within a few days, up to a week later
   6. OR
   7. If your medicine dosage was changed, such as aspirin, warfarin, or a rate control medicine, how soon do you need to see your family doctor?
      1. Within a few days, up to a week later
6. Back strain / back sprain
   1. Should you do complete bedrest initially after the injury?
      1. No (that is outdated).
   2. What should you make sure you don’t do AT ALL, in the initial days after the injury?
      1. Heavy lifting
   3. Name 2 reasons you should come back to the ER.
      1. Any 2 of inability to pee, incontinent of stool, fever, weakness or numbness in both legs.
7. Bell’s Palsy
   1. Most people fully recover after how long?
      1. 3 to 6 months. (6 months is 1 point, 3 months is 1 point, anything in between is 1 point)
   2. Can you wait a few days to start any prescription medication the physician gave you to treat this?
      1. No. (the sooner you start it, the better).
   3. How do you treat the eye on the affected side?
      1. Any of: eye drops, eye ointment, or taping eye shut with eye pad at night.
8. Broken bone, with splint
   1. Why do we put a splint on? What does the splint do?
      1. Keeps pieces of bone together, so they can heal up. Also decreases pain. [just need to say one or the other for 1 point]
   2. What is the most important thing that you can do, to speed up healing of your broken bone?
      1. Keep it elevated / up
   3. Is it okay to get a splint (fiberglass or plaster) wet?
      1. No (fiberglass is waterproof but the lining isn’t)
9. Burns
   1. When should you come back to the ER?
      1. Signs of infection to the burn (any of pus, yellow, redness spreading, you develop a fever)
   2. Should you break any blisters that come up?
      1. No. [the doctor might though.]
   3. If it is not getting infected, what should you use to dress the wound, or put on it before you apply a new bandage?
      1. Polysporin
10. Cellulitis
    1. When is the LATEST that you should see improvement in the infection, after starting antibiotics?
       1. 2 days (1 day is fine too)
    2. When should you see your family doctor that day, or come to the ER?
       1. New fever or red area getting larger/streaking up the extremity (if they say ‘getting sicker’ ask for specifics)
    3. Is it better to use the infected limb a lot or only a little, to speed up healing?
       1. As little as possible
11. Croup
    1. Do antibiotics treat / fix croup?
       1. No (it is a virus)
    2. Does croup usually get worse or better on the second night?
       1. Worse (unless dexamethasone / steroid medication was given)
    3. Name 2 reasons to come to the ER
       1. Signs of dehydration, not breathing properly, even at rest, or true lethargy.
12. Diverticulitis
    1. In addition to medication, how else should you treat the diverticulitis? What else can you do?
       1. Give the bowel a rest, by eating light foods (any of high carb, low fiber, etc)
    2. What is the PREVENTION for future attacks of diverticulitis, once the antibiotics are finished and the attack has resolved?
       1. High fibre foods
    3. Name 2 reasons you should come back to the ER
       1. fever that persists after 2 days of antibiotics, the pain is worsening instead of improving, or you develop vomiting
13. Ear infection, external (otitis externa)
    1. Can you go swimming when you have this infection?
       1. No (don’t get any water in the ear canal)
    2. How soon should your symptoms improve, with treatment?
       1. 2 days max (1 day is fine too: 1 point)
    3. Name 2 reasons to return to the ER.
       1. stiff neck, confusion or drowsiness, redness and/or swelling of the ear or the skin around your ear
14. Ear infection, inner (otitis media)
    1. If the symptoms resolve early, can you stop the antibiotics?
       1. No (finish the entire course)
    2. What usually occurs first, that then leads to an inner ear infection?
       1. A cold
    3. Name 2 reasons to return to the ER.
       1. stiff neck, confusion or drowsiness, seizure
15. Eye scratch (corneal abrasion)
    1. How long until the pain in your eye should go away completely?
       1. 24 hrs. 48 hrs max. (either is 1 point)
    2. Should you patch your eye, or put a patch over it to keep it shut?
       1. No
    3. Name 2 reasons to return to the ER, or see your ophthalmologist right away.
       1. increasing eye pain, worsening of vision, or pus coming from the eye
16. Fever in a child, uncertain cause
    1. What is the most important thing to do at home, when treating a fever?
       1. Hydration
    2. Is it safe to take ibuprofen or acetaminophen for fever?
       1. Yes
    3. Name 2 reasons to return to the ER
       1. Listless or lethargic (not making eye contact with parent), or dehydrated (less pee, no tears when crying, dry mouth and eyes), or high fever (>40.0 c or 104F)
17. Fingertip amputation
    1. How long should you keep it dry?
       1. 24 or 48 hours (either is fine for 1 pt, but 48 hours is best)
    2. What can you do to speed up the healing?
       1. Keep it elevated / up [1 pt] (polysporin = 0.5 pt)
    3. Name 2 reasons you should come back to the ER
       1. pus coming from the wound or redness around it or spreading further up your finger, fever
18. Gastroenteritis - viral
    1. What is the most important thing to do, or part of getting over, a gastroenteritis?
       1. Stay hydrated/keep hydrated
    2. Do antibiotics help treat it?
       1. No (if unsure, give zero points)
    3. Is it okay to take an anti-diarrhea medicine?
       1. Yes (1/2 point. If say yes, then ask, “when?”): 1 day or more after diarrhea began
19. Gout
    1. Which foods and drink can worsen gout, or cause an attack of gout?
       1. Rich foods (any, but can include shrimp, scallops, anchovies, wild game, gravy, etc, and to a lesser extent red meat, pork, poultry, asparagus, mushrooms, cauliflower and spinach) and alcohol
       2. What can you use for pain control, other than medicine?
          1. Bag of ice/ frozen peas
       3. Name a reason to come back to the ER
          1. fever, inability to move the joint at all despite treatment (medicine), or increasing redness around the joint despite treatment (medicine)
20. Minor head injury, with concussion
    1. What is the most important thing to avoid, in the first few weeks after a head injury?
       1. A second head injury
    2. Name 2 symptoms of a concussion
       1. Headaches, difficulties concentrating, or feeling like you are in a fog, or slow speech
    3. Name 2 reasons you should come back to the ER
       1. vomiting
       2. have a seizure
       3. develop weakness in one arm or leg
       4. develop a severe headache
       5. are confused
       6. are getting sleepier and sleepier
21. Head injury, return to play instructions
    1. In general, when can you try returning to any activity?
       1. When the symptoms are completely GONE
    2. What happens if symptoms return during the step-wise return to activity?
       1. Get re-evaluated by a doctor, before trying again
    3. About how many steps are there that the player has to achieve, without having any symptoms, before returning to play an actual game?
       1. 6 (between 4 and 8 is 1 point).
22. High blood pressure
    1. How should high blood pressure be lowered – slowly or quickly?
       1. Slowly (and safely)
    2. How soon should you see your family doctor?
       1. Within a week (preferable within a few days)
    3. Name 2 reasons you should come back to the ER
       1. chest pain, significant shortness of breath, especially when walking, a terrible headache (probably the worst of your life), visual changes, or feeling very lightheaded.
23. Kidney stones
    1. Name a medicine, or type of medicine, that works directly on the pain of kidney stones?
       1. Ibuprofen/Motrin/Advil, Naproxen/Aleve, any NSAIDs.
    2. What foods or drinks should you avoid when you have a kidney stone attack?
       1. caffeine and alcohol (1 point for either)
    3. Name 1 reason you should come back to the ER
       1. fever, burning during urination or peeing that occurs with every pee, repeated vomiting, or you stop urinating entirely over 8 hours or more (infection or retention – either are fine / 1 point)
24. Cut fixed with glue or steri-strips
    1. Should you put Polysporin on your wound that has been glued?
       1. No
    2. What should you look for when you change the dressing?
       1. Red streaking, worsening/growing redness (infection)
    3. Ask if steri-strips or glue… Is it ok to get it wet?
       1. If steri-strips, NO (will fall off, keep fully dry, dab with towel gently if gets sprayed.)
       2. If glue, you can shower, but don’t soak it (ie under water, in bath, etc)
25. Cut with stitches or staples
    1. How long should you keep it dry?
       1. 24 to 48 hours (any of 24 hours, 48 hours, 1 day, 2 days)
    2. What should you look for when you change the dressing?
       1. Red streaking, worsening/growing redness (infection)
    3. When should you get the stitches out?
       1. 5 days for face, 14 days over a joint (elbow, wrist, knee), all others 8 or 9 days (10 days is fine too) (if within a day then give full 1 point)
26. Possible miscarriage / first trimester bleeding
    1. How soon MUST you follow-up with your family doctor or obstetrician, once you leave the ER?
       1. Within a week (the sooner the better)
    2. Why must you follow-up with your fd or obstetrician? For what purpose?
       1. Blood test (beta-hcg level) and ultrasound (either is worth 1 point)
    3. Name 2 reasons you should come back to the ER
       1. new or worsening pain, particularly sharp pain in the lower abdomen, or significantly lightheaded with ongoing heavy bleeding, fever
27. Nosebleed
    1. If your nosebleed starts again, what should you do?
       1. Pinch the nose, sitting forward/head down. (if they don’t volunteer position, prompt them). (1/2 point for each)
    2. If blood is going down the back of your throat, should you swallow it?
       1. No (spit it out)
    3. How long should you pinch your nose for?
       1. 10 to 15 minutes (using a timer, don’t peak) (5 minutes minimum for 1 point)
28. Palpitations
    1. What can bring on palpitations? Name 2.
       1. Alcohol, caffeine, cold medications stress, energy drinks,
    2. What investigations or tests can you have done with the family doctor to investigate the palpitations further?
       1. Echocardiogram (echo) and/or Holter monitor (either is 1 point)
    3. Name 2 reasons to return to the ER
       1. episodes of palpitations that don’t go away in a few minutes, or develop chest pain, light headedness or faint with palpitations
29. Panic attacks
    1. In general, why do panic attacks occur? What precipitates them, or makes them happen?
       1. Baseline increase in stress (e.g. work is stressful, moving, getting married or divorced)
    2. Why are most medicines not useful for treating panic attacks?
       1. By the time the pill kicks in, the attack is mostly gone.
    3. What is a better way to treat panic attacks, that is not medicine?
       1. Reduce your stress, relaxation techniques, yoga, or stress management techniques
30. Rib fracture / broken rib or bruise
    1. What MUST you do when you have a rib fracture or bruise, which you normally do without thinking about it?
       1. Take deep breaths, and cough (1/2 point for each)
    2. If you can’t take a deep breath, or cough, because it is too painful, what must you do?
       1. Take more pain medicine, until you can.
    3. What must you look out for, which would mean you need to come back to the ER?
       1. Fever and cough (pneumonia) (either is 1 point)
31. Sciatica
    1. Should you do complete bedrest initially after the injury?
       1. No (that is old fashioned).
    2. What else may be helpful to try, in addition to medication?
       1. Physiotherapy
    3. Name 2 reasons you should come back to the ER.
       1. Any 2 of inability to pee, incontinent of stool, fever, weakness or numbness in BOTH legs.
32. Shingles
    1. Is shingles contagious?
       1. Yes
    2. Who is at risk of catching the virus from you?
       1. Pregnant women, or immunocompromised patients, or patients who haven’t had chicken pox (any = 1 point)
    3. What should you look out for, which would make you see your family doctor as soon as possible?
       1. If it is getting red/infected (need antibiotics if so).
33. Sore throat
    1. Are most sore throats caused by a virus or bacteria?
       1. Virus
    2. What can you do for pain / discomfort?
       1. Ibuprofen, acetaminophen, throat lozenges or gargling with warm salt water may also help with throat pain (any is 1 point)
    3. Name 1 reason to return to the ER
       1. unable to swallow liquids or have difficulty breathing
34. Tubal pregnancy, possible (possible ectopic pregnancy)
    1. What could happen if it is an ‘ectopic pregnancy’, or a pregnancy in the fallopian tube, or tubes, rather than the uterus, and it isn’t treated?
       1. Patient could die – fetus will continue to grow until tube ruptures (1 pt for either)
    2. How soon MUST you follow-up with your family doctor or obstetrician, once you leave the ER?
       1. 1 week, max, preferably sooner
    3. Name 2 reasons to return to the ER immediately
       1. Pain, often sharp in the lower belly, fainting or significant lightheadedness, worsening vaginal bleeding (one pad per hour for three consecutive hours), fever
35. Urinary retention
    1. In general, why do older men get urinary retention?
       1. Large prostate
    2. How long should the catheter stay in, if one was place in the ER and you were sent home with it?
       1. 1 to 2 weeks
    3. Name 2 reasons you should come back to the ER.
       1. No more drainage from the bag, new fever, back pain, blood or urine leaking from around the catheter
36. Urinary Tract Infection (UTI)
    1. What do you need to do if you had blood in your urine when you had the infection?
       1. See your family doctor for a repeat urine test, to make sure the blood is gone.
    2. When do you know that the antibiotics aren’t working? After how long of taking the antibiotics?
       1. 2 days (1 day is ok too)
    3. Name two signs that the infection is getting worse, not better.
       1. Upper back pain (new back pain is ok too), fever, lethargy (some description of lethargy is fine)
37. Vertigo (the spins)
    1. Where in your body is the problem that is causing your symptoms of vertigo, or spinning?
       1. The inner ear, or ear.
    2. What is the best way to avoid getting the vertigo, in the next week or so?
       1. Move slowly, no sudden head movements, etc
    3. Name 2 reasons you should come back to the ER
       1. changes in vision, weakness in the face or arm or leg, a headache, confusion, or getting more and more sleepy / drowsy
38. Whiplash (neck sprain)
    1. Will the pain likely be better or worse on day 2?
       1. Worse
    2. Other than medication, what else can you use for pain?
       1. Ice pack or bag of frozen peas
    3. Name 1 reason you should come back to the ER
       1. Weakness or numbness in either or both of your arms, fever
