# Supplementary material for: Speak Fast, Use Jargon, and Don’t Repeat Yourself: A Randomized Trial Assessing the Effectiveness of Online Videos to Supplement Emergency Department Discharge Instructions
Source: PLoS One. 2013 Nov 11;8(11):e77057. doi: 10.1371/journal.pone.0077057 (PMC3823877; doi:10.1371/journal.pone.0077057)
Supplement: Protocol S1 — Trial protocol. (DOC) [file pone.0077057.s003.doc]

**Speak Fast, Use Jargon, and Don’t Repeat Yourself: Using Online Videos to Supplement Emergency Department Discharge Instructions**

Primary Goal: To create a website of discharge instruction videos for patients who have been treated in the emergency department (ED).

Specific Objectives

1. To create a database of instructional videos, available on the Sunnybrook Health Sciences (SHSC) website, for patients to view after being seen in the SHSC ED.
2. To evaluate the utility and value of such a service via telephone interviews with a convenience sample of SHSC ED patients, subsequent to their ED visit.

Background and Rationale Either for themselves or a loved one, most people in society have visited an emergency department recently. In 2001 a report from the Institute for Clinical Evaluative Sciences (ICES) found that 3.7 million Ontarians made a visit to the ED that year, about one-fifth of the province’s population^1^. Given that ED crowding is ubiquitous around the world^2-5^, the time spent with the doctor is relatively short^2^. Good discharge instructions can ensure quality care, prevent poor outcomes, and lessen repeat visits in an already overwhelmed system, but the time it takes to provide them to each and every patient seen in the ED may be prohibitive^6^. Thus discharge instructions may be truncated^6^ in an effort to see more patients. Some EDs subscribe to a service that provides written, standardized discharge instructions, such as Exit-writer™^7^. These may be very helpful if (1) the hospital subscribes to them, and (2) the managing emergency physician takes the time to print them out for each patient. However the locus of control for the provision of written ED discharge instructions lies with the busy ED staff, not the patient. In addition, depending on the patient population and reading level within the population, some patients may not fully comprehend written instructions^8,9^.

Significance In a hurried, unfamiliar environment, the patient’s ability to retain the instructions told to them by the doctor may be significantly compromised. Basic learning techniques demonstrate that repetition is a key component to effective learning^10^, but in a busy ED repetition carries the cost of time, and is therefore unlikely to occur. In a recent study, half of ED patients were deficient in their comprehension of what was told to them by the ED physician, in particular in post-ED care^11^. Once home, or even out of the department, the patient may realize that they have forgotten key components of their instructions; instead of calling the ED, their family doctor, or ignoring their oversight entirely, another option would be to provide them with access to a website of ED discharge instructions, where the patient can view a short video related to their discharge diagnosis. An SHSC website would enable patients who have forgotten their ED discharge instructions, or those who would prefer to hear their instructions again (or hear more than they received in the ED), to watch a physician present their specific discharge instructions online. At the end of the video a written summary would be available for printout, or the patient could watch it again, depending on their needs and level of retention.

Current Knowledge in the Field The efficacy of video as a medium to provide effective teaching around discharge instructions is not known. This emerging technology has had exponential uptake on sites like YouTube, MySpace, Dailymotion, and Metacafe. Areas of instruction are extremely popular, such as health (“What’s in a Flu Shot”, YouTube, 131,950 views), knitting, languages, musical instruments, and even make-up application (Lauren Luke, YouTube, 56 million views). Videos for healthcare practitioners are an emerging field, and include instructional videos for procedures such as those by the New England Journal of Medicine and ProceduresConsult™^12^. However there are few websites that provide video instructions to patients. While popular TV shows like “The Dr Oz Show” have proven the public’s desire to understand their health and medical issues, no institution or site has offered high-quality, vetted discharge instruction videos by a physician, for the ED or any other health care setting.

Relationship of Project to Previous Work Our previous work includes evaluating the effectiveness of online videos in teaching medical students and residents how to perform ED procedures. In this before and after prospective study we collected data on procedures performed by trainees. The intervention was the use of one of a library of procedure videos, introduced in the “after” phase, which will be complete in March 2009. Before introducing the database of available videos we had to collate and vet procedural videos that we found online. Often a quality video for an ED procedure was not available; for these procedures we created our own videos, which were among the most highly ranked by our participants. We found that staff physicians were also eager to view the videos, which we had to deny them given that we were counting each video viewed online during the study period. Thus we created a second database for interested staff to view the videos. This lead us to wonder if patients would also appreciate a simple, short video, once their long ED stay (yet brief encounter with the physician) was over.

Approach and Work Plan The main component of the proposed innovation will be the creation and development of the scripts and videos for each of the 40 discharge diagnoses (Appendix A). Scripts have been developed by the PI and team (see below). Using a Delphi technique, summaries of each were reviewed by the team of SHSC emergency physician team members for their input. Next the video scripts were sent to 10 laypersons, to screen for comprehension. Lastly the PI will film the instructions, using the appropriate equipment for each demonstration (ie steri-strips for patients who have had facial sutures). Each video will be edited and the file compressed; length of each will be up to 5 minutes, and each will be followed by a printable discharge instruction summary sheet.

Evaluation of Outcome of Project

To assess the utility of the videos we will take two approaches. First we will assess the frequency of website use by assessing the number of website hits after giving out the website address to discharged ED patients during a two week period (the denominator will be the number of forms handed out). Next we will approach a convenience sample of 126 patients with certain ED discharge diagnoses for follow-up within five days of the index ED visit, consenting them to a follow-up phone call. We will use randomization by patient to randomize patients to receiving the website address or not. In follow-up phone calls we will confirm who viewed the video (which can also be confirmed with the website counter), test understanding of their discharge instructions using a standardized telephone interview (three questions created specifically for the 38 diagnoses of interest – two are not amenable to questions), and, in those patients who viewed a video, ask them to rate the utility of the discharge video.

Outcomes of Interest

1. Rate of Use: The rate of use will be recorded by the number of “hits” the website receives. The denominator will be the number of people given the website address by the research assistant (RA) during a two-week preliminary period (this will also allow website issues to be addressed before the second phase of video evaluation).
2. Patient-Rated Utility: In discharged ED patients who viewed a video, what was the median rating (Likert scale 1 to 5) of (i) the usefulness of the videos in understanding of their discharge instructions, and (ii) overall usefulness?
3. Patient comprehension of discharge instructions: As assessed by three key, pilot-tested questions specific to each of 38 diagnoses (Appendix B), was the test success rate in discharged patients who saw a video significantly better than in those who did not view an online video?

Data Collection A trained RA will approach patients who have one of the 38 pre-defined discharge diagnoses during the six-week study period. Inclusion criteria will include access to the internet in the next five days, the ability to find the website, and a telephone. In eligible patients, the RA will obtain consent to contact the patient between one and five days post ED visit, and randomize the patients to either the video group (offering them the website address) or standard care, according to a randomized schedule of 140 patients (enrollment will stop after 126 if all are able to be contacted). Patients will be contacted by the RA and asked a standardized set of three questions about their specific diagnosis and discharge instructions, as well as asked to rate the usefulness of the video, if in the video group. Data will be entered into an Excel file by the RA.

Timeline The main component of the proposed innovation is the development of the discharge instruction videos. Currently all discharge video scripts have been proofed, and now require filming. Once filming is completed, and the videos are posted to the SHSC Department of Emergency Services website (and testing is completed), the two week website use period can commence **[goal commencement date: July 4/11]**. Next, enrollment of 126 patients, at a conservative five patients/day, will require 30 days (six weeks of weekdays) **[goal commencement date: July 18/11]**. Data cleaning and analysis will take several months, and manuscript preparation one to two months.

Team The team consists of the following: (1) PI, a clinician scientist and an assistant professor at the University of Toronto, as well as a practicing emergency physician at SHSC; (2) Michael Feldman MD FRCPC, a practicing emergency physician at SHSC who has specialized IT skills, and is the head of IT in the Department of Emergency Services at SHSC; and (3) three additional co-applicants who created and reviewed the planned discharge scripts. All three are practicing emergency physicians at SHSC, and were selected based on number of years of practice: Michael Brzozowski MD FRCPC (10-15 years), Michael McDonnell MD FRCPC (5-10 years), and Laurie Mazurik MD FRCPC (>20 years). The PI has access to statisticians at ICES. The videos will be filmed and edited by SHSC Media and Print services. Dr Feldman is in charge of the Department of Emergency Services SHSC website and will approve the posting the videos, as well as oversee the site for problems.

Analysis Descriptive statistics will be used where appropriate. For the rate of online video use, proportions with 95% confidence intervals will be used. This rate will be compared to similar start-up websites. The patient rated utility will be reported using median and IQR. For the RCT we will analyze by intention-to-treat, but also perform a sensitivity analysis to assess the efficacy of the videos. We will compare the scores overall, and in five pre-selected discharge diagnosis (cast care, suture care, kidney stones, chest pain NYD, and allergic reaction) using a t-test. A sample size calculation reveals that we need 16 patients per group to show a difference of 1 point between test scores (maximum score of 3) using 1.0 sd. To detect a difference of half a point, 63 patients per group are needed.

Impact and Knowledge Translation This project represents a major innovation, which could increase SHSC’s visibility in Canada and the world. No other centre offers this service, thus SHSC would be the first to provide online discharge instructions, offering a complete cycle of care to their patients. Other centres would likely be quick to emulate this format, but SHSC would be the first to offer it. The project could easily be transferred to other institutions, and/or expanded to other health care settings. It is itself a knowledge translation project, for patients. In addition it may improve patient safety, decrease the number of repeat ED visits and unnecessary family doctor visits, and it offers patients an opportunity to learn more about their own health, enabling them to take control of the final phase of their ED visit.

Sustainability SHSC has an established website, and on that website is a departmental website for Emergency Services. The physician in charge of this site is a co-applicant on this grant, and there is ongoing funding to maintain the website. The grant has been approved by the director of the Department of Emergency Services.

Appendix A. List of 40 common or important emergency department discharge diagnoses, for which a discharge video will be filmed and posted on the SHSC Department of Emergency Services website (38 are appropriate for 3 questions)

1. Viral gastroenteritis / vomiting and diarrhea

2. Urinary Tract Infection

3. Asthma exacerbation/attack

4. Abscess, I&D

5. Cellulitis

6. Atrial fibrillation

7. Ankle sprain

8. Bell’s Palsy

9. Shingles

10. Rib fracture or contusion (broken or bruised ribs)

11. Burns

12. Panic attacks

13. Back strain

14. Whiplash/neck strain

15. Croup

16. Urinary retention

17. Gout attack

18. High blood pressure, out of control

19. Sciatica

20. Diverticulitis, uncomplicated

21. Kidney stone

22. Laceration/cut, stitches or staples used

23. Laceration/cut, glue or tape used

24. Fracture/broken bone, with splint

25. Miscarriage, possible

26. Head injury, minor, with concussion

27. Allergic reaction

28. Vertigo (peripheral) or “the spins”

29. Nosebleed

30. Fingertip amputation

31. Palpitations

32. Pharyngitis (sore throat)

33. Ear infection, inner – Otitis media

34. Ear infection, outer – Otitis externa

35. Possible ectopic pregnancy (tubal pregnancy)

36. Fever in a child

37. Head injury, minor, with return to play guidelines

38. Chest pain, not yet diagnosed

39. Corneal abrasion (eye scratch)

40. How to find a family doctor

Appendix B. Standardized telephone interview questions

“As you know, you were diagnosed with a ___ when you were in the ER.”

1. Viral Gastroenteritis
   1. What is the most important thing to do, or part of getting over, a gastroenteritis?
      1. Stay hydrated/keep hydrated
   2. Do antibiotics help to treat a viral gastroenteritis?
      1. No (if unsure, zero points)
   3. Is it okay to take an anti-diarrhea medicine?
      1. Yes (1/2 point. If say yes, then ask, “when?”): 1 day or more after diarrhea began
2. Urinary Tract Infection (UTI)
   1. What do you need to do if you had blood in your urine when you had the infection?
      1. See your family doctor for a repeat urine test, to make sure the blood is gone.
   2. When do you know that the antibiotics aren’t working? In other words, after how long of taking the antibiotics should the symptoms have disappeared, at the latest?
      1. 2 days (1 day is ok too)
   3. Name two signs that the infection is getting worse, not better. [1/2 pt each, max 1 pt]]
      1. Upper back pain (new back pain is ok too), fever, lethargy (some description of lethargy is fine)
3. Asthma Exacerbation
   1. Which puffer should you take when your breathing and/or wheezing gets worse, during an asthma attack? [any is 1 pt]
      1. Blue puffer, ventolin puffer, atrovent puffer, white & green puffer.
   2. How often and how much should you be taking the blue puffer (ventolin) in the first few days after you are discharged from the ER?
      1. 2 puffs (‘a few’ puffs is okay too) every 4 hours (or every 6 hours) – ½ pt each. “A lot” = ½ pt (ask them to be more specific)
   3. When should you come back to the ER?
      1. Requiring > 2 puffs less than every 4 hrs, can’t speak full sentences, breathing labored. [Any is 1 pt]
4. Abscess incision & drainage
   1. If there is drainage (fluid) coming from the wound, getting your bandages dirty, is this good or bad?
      1. Good (it is draining)
   2. Why is the cotton gauze put inside the wound?
      1. To keep the wound open, so it can drain.
   3. How long should you keep the packing in?
      1. 2 days (1 day is fine too, as is 3 days).
5. Cellulitis
   1. When is the LATEST that you should see improvement in the infection, after starting antibiotics?
      1. 2 days (1 day is fine too)
   2. When should you see your family doctor that day, or come to the ER?
      1. New fever or red area getting larger/streaking up the extremity (if they say ‘getting sicker’ ask for specifics)
   3. Is it better to use the infected limb a lot or only a little, to speed up healing?
      1. As little as possible
6. Atrial Fibrillation [Questions asked depend on treatment strategy]
   1. What is the big concern about atrial fibrillation? What can happen to patients who have atrial fibrillation?
      1. A stroke
   2. *If you were put on warfarin*, does it matter which pharmacy you take the prescription to?
      1. Yes. Lots of interactions with warfarin
   3. OR
   4. [*if not put on warfarin*, as the following question] How is a stroke prevented?
      1. Aspirin or warfarin
   5. *If you were put on a new medicine*, such as aspirin, warfarin, or a rate control medicine, how soon do you need to see your family doctor?
      1. Within a few days (up to a week later, max)
   6. OR
   7. *If your medicine dosage was changed*, such as aspirin, warfarin, or a rate control medicine, how soon do you need to see your family doctor?
      1. Within a few days, up to a week later
7. Ankle Sprain
   1. How do you decide when you can walk on your ankle?
      1. If it hurts a lot, don’t. (listen to your ankle, let your ankle tell u, etc)
   2. What is the most important position for your ankle or leg when you are recovering?
      1. Elevated
   3. How long will it take to heal?
      1. Weeks (2 weeks or less, zero points). Up to 6 months to heal completely (only need to say ‘weeks’ or 3 weeks and up to get a point)
8. Bell’s Palsy
   1. Most people fully recover after how long?
      1. 3 to 6 months. (6 months is fine, 3 months is fine, anything in between is fine)
   2. Can you wait a few days to start any prescription medication the physician gave you to treat this?
      1. No. the sooner you start it, the better.
   3. How do you treat the eye on the affected side?
      1. Any of: eye drops, eye ointment, or taping eye shut with eye pad at night.
9. Shingles
   1. Is shingles contagious?
      1. Yes
   2. Who is most at risk of catching the virus from you?
      1. Pregnant women, or immunocompromised patients, or patients who haven’t had chicken pox [any = 1 pt]
   3. Why is it often so painful?
      1. Because the infection is inside a nerve (and a nerve transmits pain).
10. Rib fracture / broken rib or bruise
    1. What MUST you do when you have a rib fracture or bruise, which you normally do without thinking about it?
       1. Take deep breaths, and cough [1/2 pt each. If only say one, prompt for another]
    2. If you can’t take a deep breath, or cough, because it is too painful, what must you do?
       1. Take more pain medicine, until you can.
    3. What must you look out for, which would mean you need to come back to the ER?
       1. Fever and cough (pneumonia)
11. Burn
    1. When should you come back to the ER?
       1. Signs of infection to the burn (pus, yellow, redness spreading, u develop a fever…)
    2. Should you break any blisters that come up?
       1. No. [the doctor might though.]
    3. If it is not getting infected, what should you use to dress the wound, or put on it before you apply a new bandage?
       1. Polysporin
12. Panic attacks
    1. In general, why do panic attacks occur? What precipitates them, or makes them happen?
       1. Baseline increase in stress.
    2. Why are most medicines not useful for treating panic attacks?
       1. By the time the pill kicks in, the attack is mostly gone.
    3. What is a better way to treat panic attacks, which is not medicine?
       1. Reduce your stress, relaxation techniques, yoga, stress management techniques, etc
13. Back strain / back sprain
    1. Should you do complete bedrest initially after the injury?
       1. No, that is old fashioned.
    2. What should you make sure you don’t do AT ALL, in the initial days after the injury?
       1. Heavy lifting
    3. Name 2 reasons you should come back to the ER.
       1. Any 2 of inability to pee, incontinent of stool, fever, weakness or numbness in BOTH legs. [1/2 pt each, max 1 pt]
14. Whiplash
    1. Will the pain likely be better or worse on day 2?
       1. Worse
    2. Other than medication, what else can you use for pain?
       1. Ice pack, bag of frozen peas, etc
    3. Name 1 reason you should come back to the ER
       1. Weakness or numbness in either or both of your arms, fever [either is 1 pt]
15. Croup
    1. Do antibiotics treat / fix croup?
       1. No, it is a virus
    2. Does croup usually get worse or better on the second night?
       1. Worse [unless dex / steroid was given]
    3. Name 2 reasons to come to the ER [1/2 pt each, max 1 pt]
       1. Signs of dehydration, not breathing properly, even at rest, or true lethargy.
16. Urinary retention (simple)
    1. In general, why do older men get urinary retention?
       1. Large prostate
    2. How long should the catheter stay in, if one was place in the ER and you were sent home with it?
       1. 1 to 2 weeks (5 days is fine)
    3. Name 2 reasons you should come back to the ER. [½ pt each, max 1 pt]
       1. No more drainage from the bag, new fever, back pain, blood or urine leaking from around the catheter
17. Gout
    1. Which foods and drink can worsen gout, or cause an attack of gout?
       1. Rich foods (any, but can include shrimp, scallops, anchovies, wild game, gravy, etc, and to a lesser extent red meat, pork, poultry, asparagus, mushrooms, cauliflower and spinach) and alcohol
    2. What can you use for pain control, other than medicine?
       - 1. Bag of ice/ frozen peas
    3. Name a reason to come back to the ER
       - 1. fever, inability to move the joint at all despite treatment (medicine), increasing redness around the joint despite treatment (medicine)
18. High blood pressure
    1. How should high blood pressure be lowered – slowly or quickly?
       1. Slowly (and safely)
    2. How soon should you see your family doctor?
       1. WITHIN a week (preferable within a few days) [1 pt for within a few days, 0.5 pt if say 1 week.]
    3. Name 2 reasons you should come back to the ER [½ pt each, max 1 pt]
       1. chest pain, significant shortness of breath, especially when walking, a terrible headache (probably the worst of your life), and visual changes. Or feeling very lightheaded.
19. Sciatica
    1. Should you do complete bedrest initially after the injury?
       1. No, that is old fashioned.
    2. What else may be helpful to try, in addition to medication?
       1. Physio
    3. Name 2 reasons you should come back to the ER. [½ pt each, max 1 pt]
       1. Any 2 of inability to pee, incontinent of stool, fever, weakness or numbness in BOTH legs.
20. Diverticulitis
    1. In addition to medication, how else should you treat the diverticulitis? What else can you do?
       1. Give the bowel a rest, by eating light foods (high carb, low fiber, etc)
    2. What is the PREVENTION for future attacks of diverticulitis, once the antibiotics are finished and the attack has resolved?
       1. High fibre foods
    3. Name 2 reasons you should come back to the ER [½ pt each, max 1 pt]
       1. fever that persists after 2 days of antibiotics, the pain is worsening instead of improving, or you develop vomiting
21. Kidney stones
    1. Name a medicine, or type of medicine, that works directly on the pain of kidney stones?
       1. Ibuprofen, nsaids, aleve/naproxen, etc.[any is 1 pt]
    2. What drinks should you avoid when you have a kidney stone attack?
       1. caffeine and alcohol [any = 1 pt]
    3. Name 1 reason you should come back to the ER
       1. fever, burning during urination or peeing that occurs with every pee, repeated vomiting, or you stop urinating entirely over 8 hours or more (infection or retention – either are fine answers)
22. Cut with stitches or staples
    1. How long should you keep it dry?
       1. 24 to 48 hours (any of 24 hrs, 48 hrs, 1 day, 2 days)
    2. What should you look for when you change the dressing?
       1. Red streaking, worsening/growing redness (infection)
    3. How often should you apply polysporin or similar ointment, to the wound?
       1. Every day, or even twice a day
23. Cut fixed with glue or steri-strips
    1. Should you put polysporin on your wound that has been glued?
       1. No
    2. What should you look for when you change the dressing?
       1. Red streaking, worsening/growing redness (infection)
    3. Ask if steristrips or glue… Is it ok to get it wet?
       1. *If steristrips*, NO, not at all. Keep fully dry, dab with towel gently if gets sprayed.
       2. *If glue*, you can shower, but don’t soak it (ie under water, in bath, etc)
24. Broken bone, with splint
    1. Why do we put a splint on? What does the splint do?
       1. Keeps pieces of bone together, so they can heal up. Also decreases pain. [just need to say one or the other for 1 pt]
    2. What is the most important thing that you can do, to speed up healing of your broken bone?
       1. Keep it elevated
    3. Is it okay to get a splint (fiberglass or plaster) wet?
       1. No
25. First trimester bleeding / possible miscarriage
    1. How soon MUST you followup with your family dr or ostetrician, once you leave the ER?
       1. 3 days, max
    2. Why must you followup with your fd or obstetrician? What will they do?
       1. Blood test (beta hcg level) and ultrasound [either is 1 pt]
    3. Name 2 reasons you should come back to the ER [½ pt each, max 1 pt]
       1. new pain, particularly sharp pain in the lower abdomen, or significantly lightheaded with ongoing heavy bleeding, fever
26. Minor head injury, with concussion
    1. What is the most important thing to avoid, in the first few weeks after a head injury?
       1. A second head injury
    2. Name 2 symptoms of a concussion [½ pt each – max 1 pt]
       1. Headaches, difficulties concentrating, or feeling like you are in a fog, or slow speech, etc
    3. Name 2 reasons you should come back to the ER [½ pt each – max 1 pt]
       1. vomiting,
       2. have a seizure,
       3. develop weakness in one arm or leg,
       4. develop a severe headache,
       5. are confused, or
       6. are getting sleepier and sleepier
27. Allergic Reaction
    1. When should you use your epipen?
       1. Tongue, mouth, or throat swelling, or difficulties breathing [either is 1 pt]
    2. How long do you need to take diphenhydramine, or benedryl, for, after you leave the ER? This includes non-drowsy versions of this medication, like “Reactine” or “Claritine”.
       1. 48 hours, or 2 days
    3. If you are uncertain whether you need it, should you use your epipen?
       1. Yes
28. Vertigo
    1. Where is the problem, which has caused your symptoms of vertigo, or spinning?
       1. The inner ear, or ear.
    2. What is the best way to avoid getting the vertigo, in the next week or so?
       1. Move slowly, no sudden head movements, etc
    3. Name 2 reasons you should come back to the ER [½ pt each – max 1 pt]
       1. changes in vision, weakness in the face or arm or leg, a headache, confusion, or getting more and more sleepy / drowsy
29. Nosebleed
    1. If your nosebleed starts again, what should you do?
       1. Pinch the nose, sitting forward. (if they don’t volunteer position, prompt them).
    2. If blood is going down the back of your throat, should you swallow it?
       1. No! spit it out
    3. How long should you pinch your nose for?
       1. 10 to 15 minutes, USING A TIMER. DON’T PEAK
30. Fingertip amputation
    1. How long should you keep it dry?
       1. 24 or 48 hours (either is fine for 1 pt, but 48 hrs is best)
    2. What can you do to speed up the healing?
       1. Keep it elevated [1 pt] (polysporin = 0.5 pt)
    3. Name 2 reasons you should come back to the ER [½ pt each, max 1 pt]
       1. pus coming from the wound or redness around it or spreading further up your finger, fever
31. Palpitations
    1. What can bring on palpitations? Name 2. [½ pt each, max 1 pt]
       1. Alcohol, caffeine, stress, energy drinks, cold medications
    2. What investigations or tests can you have done with the family doctor to investigate the palpitations further?
       1. Echocardiogram (echo) and/or holter monitor (either is 1 pt)
    3. Name 2 reasons to return to the ER
       1. episodes of palpitations that don’t go away in a few minutes, or develop chest pain, light headedness or faint with palpitations
32. Sore throat
    1. Are most sore throats caused by a virus or a bacteria?
       1. Virus
    2. What can you do for pain / discomfort?
       1. Ibuprofen, acetaminophen, Throat lozenges or gargling with warm salt water may also help with throat pain [any is 1 pt]
    3. Name 1 reason to return to the ER
       1. unable to swallow liquids or have difficulty breathing
33. Inner ear infection (otitis media)
    1. If the symptoms resolve early, can you stop the antibiotics?
       1. No. Finish the entire course
    2. What usually occurs first, that then leads to an inner ear infection?
       1. A cold
    3. Name 2 reasons to return to the ER] . [1/2 pt each, max 1 pt]
       1. stiff neck, confusion or drowsiness, seizure
34. External ear infection (otitis externa)
    1. Can you go swimming when you have this infection?
       1. No. Don’t get any water in the ear canal
    2. How soon should your symptoms improve, with treatment?
       1. 2 days
    3. Name 2 reasons to return to the ER. [1/2 pt each, max 1 pt]
       1. stiff neck, confusion or drowsiness, redness and/or swelling of the ear or the skin around your ear
35. Early pregnancy bleeding – possible ectopic pregnancy
    1. What could happen if it is an ‘ectopic pregnancy’, or a pregnancy in the fallopian tube, or tubes, rather than the uterus, and it isn’t treated?
       1. Patient could die – fetus will continue to grow until tube ruptures
    2. How soon MUST you followup with your family dr or ostetrician, once you leave the ER?
       1. 3 days, max
    3. Name 2 reasons to return to the ER immediately [1/2 pt each, max 1 pt]
       1. Pain, often sharp in the lower belly, fainting or significant lightheadedness, worsening vaginal bleeding (one pad per hour for three consecutive hours), fever
36. Fever in a child, uncertain cause
    1. What is the most important thing to do at home, when treating a fever?
       1. Hydration
    2. Is it safe to take ibruprofen or acetaminophen for fever?
       1. Yes
    3. Name 2 reasons to return to the ER [1/2 pt each, max 1 pt]
       1. Listless or lethargic (not making eye contact with parent), or dehydrated (less pee, no tears when crying, dry mouth and eyes), or high fever (>40.0 c or 104F)
37. Head injury, return to play instructions
    1. In general, when can you try returning to any activity?
       1. When the symptoms are completely GONE
    2. What happens if symptoms return during the step-wise return to activity?
       1. Get re-evaluted by a doctor, BEFORE trying again
    3. About how many steps are there that the player has to achieve, without having any symptoms, before returning to play an actual game?
       1. 6 [between 4 and 8 is 1 pt.
38. Chest pain, uncertain cause
    1. *Not appropriate for questions / testing*
39. Corneal abrasion
    1. How long until the pain in your eye should go away completely?
       1. 24 hrs. 48 hrs max.
    2. Should you patch your eye, or put a patch over it to keep it shut?
       1. No
    3. Name 2 reasons to return to the ER, or see your ophthalmologist right away.
       1. increasing eye pain, worsening of vision, or pus coming from the eye
40. How to find a family doctor
    1. *Not appropriate for questions / testing*
